# Subjective tinnitus patients with normal pure-tone hearing still suffer more informational masking in the noisy environment

**DOI:** 10.3389/fnins.2022.983427

**Published:** 2022-08-24

**Authors:** Mengyuan Wang, Jinjun Liu, Lingzhi Kong, Yixin Zhao, Tongxiang Diao, Xin Ma

**Affiliations:** ^1^School of Psychology, Beijing Normal University, Beijing, China; ^2^School of Communication Sciences, Beijing Language and Culture University, Beijing, China; ^3^Department of Otolaryngology, Head and Neck Surgery, People’s Hospital, Peking University, Beijing, China

**Keywords:** subjective tinnitus, tinnitus mechanisms, informational masking, energetic masking, spatial cues

## Abstract

Subjective tinnitus patients experience more hearing difficulties than normal peers in complex hearing environments, even though most of these patients have normal pure-tone hearing thresholds. Using speech recognition tasks under different masking conditions can provide insight into whether the effects of tinnitus are lateralized and the mechanisms behind the effects. By simulating sound field recordings, we obtain a target speech sentence that can be perceived as presented on one side and noise or speech masking with or without spatial separation from it. Our study used the virtual sound field technique to investigate the difference in speech recognition ability between chronic subjective tinnitus patients and a normal-hearing control group under the four masking conditions (speech-spectrum noise masking or two-talker speech masking, with or without perceived spatial separation). Experiment 1 showed no differences for target speech perceived location (left or right), which rules out a lateralization of the effect of tinnitus patients. Experiment 2 further found that although tinnitus patients had weaker performance than normal people in very complex auditory scenarios, when the spatial cue of the target speech exists, they can make good use of this cue to make up for the original processing disadvantage and achieve a similar performance as the normal-hearing group. In addition, the current study distinguished the effects of informational masking and energetic masking on speech recognition in patients with tinnitus and normal hearing. The results suggest that the impact of tinnitus on speech recognition in patients is more likely to occur in the auditory center rather than the periphery.

## Introduction

Subjective tinnitus is a perception of phantom sound heard by a person in the absence of any external physical stimulation (Roberts et al., 2010). The prevalence of tinnitus in adults varies across various studies (McCormack et al., 2016), ranging from 5.1 to 42.7%, which is likely influenced by the phrasing of the question. Most often, subjective tinnitus is associated with aging, hearing loss, head trauma and noise exposure (Baguley et al., 2013). In addition to hearing-loss peers, approximately 10% of tinnitus patients have normal hearing sensitivity, defined as pure-tone thresholds less than 25 dB HL at 0.25, 0.5, 1, 2, 4, and 8 kHz (Theodoroff and Folmer, 2013). For tinnitus patients with hearing loss, the mechanisms of their tinnitus can usually be related to a functional loss of hair cells in the inner ear and neuronal activities in the auditory nervous system (Noreña, 2015; Ma et al., 2021). However, does the central auditory system function abnormally in tinnitus patients with normal pure-tone hearing? At present, some researchers have explored the evidence of chronic tinnitus through cortical auditory evoked potentials, brain signal variability and delayed memory (Cardon et al., 2022), but the underlying mechanism is still unclear. Furthermore, is there any effect on speech recognition performance under different masking conditions? Thus, our study included individuals with tinnitus and normal hearing to explore the effect of subjective tinnitus on speech recognition in masking.

Speech recognition, especially in reverberant noisy environments, is an important ability in people’s daily lives. Speech recognition in noise (SIN) is a complicated multifaceted process, including bottom-up sensory encoding of target speech from the peripheral to the central auditory system and compensatory sensorimotor integration, supported by higher-level cognitive functions such as working memory and selective attention (Du et al., 2014; Coffey et al., 2017; Zhang et al., 2021). Many previous studies have found that people with hearing difficulties, such as elderly individuals (Anderson et al., 2013), children (Litovsky, 2005) and patients with a history of idiopathic sudden sensorineural hearing loss (ISSHL) (Diao et al., 2022), have poorer performance with SIN. There are two types of interference in such a process that make speech recognition difficult: energetic masking and informational masking (Freyman et al., 1999; Wu et al., 2005; Villard and Kidd, 2019). Energy masking is the disturbance of maskers that induces neural activity in the auditory periphery and reduces the reception of target information. Informational masking is the interference generated by the information contained in the masking sound, mainly affecting higher-level cognitive processing. Due to the different ways in which the two types of masking affect speech recognition, people can use some cognitive cues (such as spatial separation from target to masking) to release from information masking greatly but not energy masking significantly. Freyman et al. (1999), in his classical experimental paradigm, produced energy masking through noise and used two or more talkers’ speaking to cause informational masking.

At the same time, it is true that some tinnitus patients suffer from hearing loss or that older individuals report hearing difficulty in their daily lives (Vielsmeier et al., 2016; Ivansic et al., 2017). For patients with normal hearing, does tinnitus alone have an impact on their speech recognition? Several studies have involved SIN in tinnitus patients with normal hearing. However, there is no consistent conclusion among studies on the performance of tinnitus patients. Some of these studies have found that the speech recognition performance of tinnitus patients is worse than that of normal people of the same age (Huang et al., 2007; Hennig et al., 2011; Ryu et al., 2012; Jain and Sahoo, 2014; Moon et al., 2015; Gilles et al., 2016). Other studies have found that tinnitus patients have lesser performances in speech recognition only under individual task conditions and no significant difference from the normal population under other conditions (Tai and Husain, 2018; Zeng et al., 2020). Some studies have evaluated the association of factors such as tinnitus loudness, THI score, otoacoustic emissions, auditory brain stem responses (ABR) and the ability to discriminate the sound spectrum with SIN performance and found no significant correlation (Gilles et al., 2016; Tai and Husain, 2018). Notably, two studies have found differences in speech recognition performance in both ears of tinnitus patients (Moon et al., 2015; Tai and Husain, 2018). Such results, which are different from other studies, seem to predict the possibility of the lateralization of auditory processing in patients with tinnitus. The experimental paradigms used in these studies are quite different.

There may be two reasons for the discrepancies in previous research results. On the one hand, tinnitus is a very heterogeneous symptom, and there are many individual differences among patients. In previous studies, the type of tinnitus, the duration of symptoms, and the age of the patients were different. These factors may be the reasons for individual differences and affect the performance of speech recognition tasks. On the other hand, different experimental paradigms have various sensitivities to differences in listener auditory processing and speech recognition performance. For example, a measure named QuickSIN (Quick Speech in Noise test), which syntactically corrects sentences with low semantic cues, is more sensitive to performance differences between normal-hearing and hearing-impaired groups than the BKB-SIN (Bamford–Kowal–Bench SIN) and the hearing in noise test (HINT), which use meaningful sentences (Wilson et al., 2007). Additionally, previous studies found that attention, fatigue and other factors are considered important mediating factors between tinnitus and its impact (Andersson and Westin, 2008), and subjects need to invest more cognitive resources in some experimental paradigms but less in others. Therefore, the effect of tinnitus on speech recognition remains understudied, contributing to a lack of understanding of the mechanisms of tinnitus with normal hearing.

In summary, the current study focused on two issues worth investigating: whether there are interaural differences in speech recognition performance in tinnitus patients with normal hearing and whether tinnitus patients with normal hearing have difficulty in speech recognition compared to healthy adults. To reduce the heterogeneity of the tinnitus patient population, we focused the study population on relatively young patients with chronic subjective tinnitus who had symptoms for more than 6 months and tried to select patients with bilateral tinnitus. We investigated the patients’ SIN performance under speech-spectrum noise and two-talker speech using a perceived spatial separation paradigm (Wu et al., 2005). Additionally, spatial separation between target and interference is an important cognitive cue for listeners in SIN recognition, so we considered spatial separation an experimental factor. In Experiment 1, we sought to verify the existence of interaural differences of the patients by comparing the SIN performance on one side and the other as the target sound is perceived. In Experiment 2, we compared speech recognition performance in tinnitus patients with normal hearing and healthy adults. In Experiment 2, we compared speech recognition performance in tinnitus patients with normal hearing and healthy adults with matched age, gender and education level. This is conducive to more clearly showing the effect of tinnitus and provides a scientific research basis for exploring the mechanisms of tinnitus with normal hearing.

## Experiment 1

### Materials and methods

#### Participants

Eight tinnitus patients (3 females and 5 males, mean age = 29.2 years) who met the following criteria participated in our research. They all had bilateral subjective tinnitus that persisted for more than 6 months, with normal hearing thresholds (≤20 dB HL) at audiometric frequencies from 250 to 8,000 Hz (the only exception was one patient who had a hearing threshold of 30 dB at 8,000 Hz). Institutional Review Board of the Faculty of Psychology, BNU approved the study (202206260076) and all participants provided written informed consents.

#### Apparatus and stimuli

A set of special Chinese nonsense sentences the same as the sentences used by Wang et al. (2018) were used as the target sentences. The sentences were semantically anomalous but syntactically correct, and their English translations are partially similar to the English nonsense sentences used by Freyman et al. (1999). For example, the English translation of a sentence is “A frog always sets up your cup.” Each sentence consists of six Chinese words, with two characters for each word and one syllable for each character, and has three keywords within them: subject, predicate, and object. Target sentences were spoken by a young female talker (Talker A) at a stable rate, and the duration of a sentence was approximately 2 s.

The speech masker was a 47-s loop of digitally combined continuous recordings of Chinese nonsense sentences spoken by two different young female talkers (Talkers B and C). The noise masker was a stream of steady-state speech-spectrum noise, whose spectrum was representative of the average spectrum of the target sentences.

The acoustic signals were recorded by two microphones placed on the two sides of a simulated head and presented binaurally through ATH-MSR7 headphones driven by a desktop computer. When recording sound stimuli, we put the target speech on one side (90° relative to the front of the simulated head) and then put the masking on the same side or opposite side of the target speech. In this way, we controlled the contents and loudness of the acoustic signals that participants received the same on the two sides, and participants perceived the target on only one side according to the “precedence” effect (although the sounds were delivered to each side) (Li et al., 2005). Maskers were perceived in the same way so we could control whether there was a spatial separation of targets and maskers. Target-speech sounds were presented at an SPL of 56 dBA. The SPLs of the maskers were adjusted to produce five signal-to-noise ratios (SNRs) (–12, –8, –4, 0, 4 dB), and four consequent SNRs were used according to the performance of a participant in practice trials.

#### Design and procedure

Experiment 1 had four within-subject factors: masker type (noise masker, speech masker), the side from which the participants perceived the target (left, right), perceived spatial separation (colocation, separation) and SNRs (four consequent levels from –12, –8, –4, 0, 4 dB). Fifteen target sentences were used in each condition, and 480 trials were used for each participant.

Stimuli were presented by *Presentation* program. In each trial, the participant pressed the “space” bar on the keyboard to start the masker sound. Approximately 1 s later, a single target sentence was presented with the masker. Then, the masker was gated off as soon as the target ended. Participants were asked to vocally repeat the whole target sentence they heard as much as possible soon after the acoustic signals stopped. The number of correctly identified syllables in the keyword was recorded by the experimenter. There was a set of practice trials of each condition before the formal experiment.

#### Data analysis

A logistic psychometric function (Eq. 1) was fit to the mean data across the four SNR levels for each participant, where y is the probability of the correct identification of keywords, x is the SNR corresponding to y, μ is the SNR corresponding to 50% correct on the psychometric function, and σ determines the slope of the psychometric function:


(1) $$y=\frac{1}{1+e^{-\sigma \left(x-\mu \right)}}$$

### Results

**FIGURE 1:**
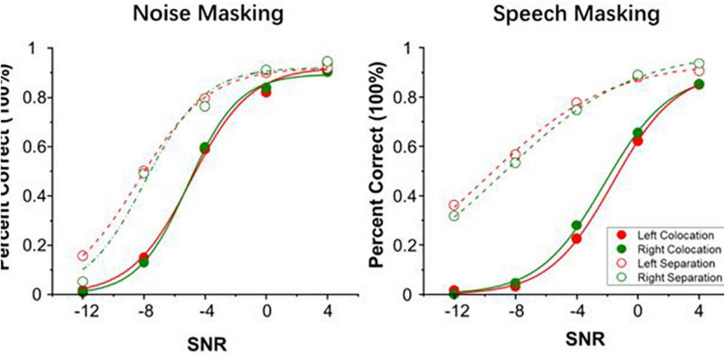
Group mean percent of correct as a function of signal-to-noise ratio (SNR) in noise masking condition **(left panel)** and speech masking condition **(right panel)**. Red lines represent when targets were perceived from the left. Green lines represent when targets were perceived from the right.

Figure 1 shows the psychometric functions of SIN perception performance on both target-perceived sides under two types of masking conditions, noise masking and speech masking. Through these functions, we could calculate the SIN perception threshold values of 50% correct (μ, in dB) in the SNR for tinnitus patients under each condition. Paired sample *t*-tests showed that the SIN perception thresholds of the two sides from which the participants perceived the target were not significant under each condition (noise masking colocation, *t* = 0.243, *p* = 0.815; speech masking colocation, *t* = 0.866, *p* = 0.415; noise masking separation, *t* = 0.159, *p* = 0.879; speech masking separation, *t* = 0.012, *p* = 0.997). These results did not support interaural differences in speech recognition in patients with tinnitus and normal hearing, suggesting that the performance is consistent between the left and right sides.

### Discussion

The results of the current study support the absence of interaural differences in speech perception performance in patients with tinnitus, consistent with most previous studies on SIN perception in tinnitus patients and normal-hearing adults. As mentioned above, two previous studies have found interaural differences in speech recognition in patients with tinnitus (Moon et al., 2015; Tai and Husain, 2018). Moon et al. (2015) considered unilateral tinnitus patients with hearing loss or normal hearing and found that tinnitus-affected ears showed poorer SRTs than non-tinnitus ears in SIN performance. There are differences in the groups and questions that Moon’s study and our study focused on, so the differences in results are explainable. More notably, Study of Tai and Husain (2018) found a right-ear advantage for SIN recognition in patients with non-lateralized tinnitus and normal hearing but not in the control group. Some researchers believe that there is truly a right-ear advantage in SIN perception because the left hemisphere dominates speech and language, and conduction of the right ear can efficiently conduct signals to the left hemisphere (Kimura, 2011). Past research on older adults also found a right-ear advantage in their SIN recognition, and the advantage seemed to increase with normal aging and age-related hearing loss (Jerger et al., 1994; Roup, 2011). In the view of some researchers, right-ear advantage, or in other words left-ear disadvantage, was due to a decline in cognitive functions or a loss in efficiency of interhemispheric transfer at the corpus callosum (Jerger et al., 1994; Roup, 2011). The participants from study of Tai and Husain were 43.86 years on average, and they were more likely to be affected by age-related difficulty in speech perception than patients in the current study. Therefore, the current results should be plausible for the group of participants in this study, the younger population with bilateral tinnitus and normal hearing.

Based on the above results and previous research viewpoints, we had sufficient reasons to believe that there was no interaural difference in speech recognition among the tinnitus patients participating in the experiment. Therefore, the possibility of SIN recognition lateralization would no longer be considered in Experiment 2.

## Experiment 2

### Materials and methods

#### Participants

##### Tinnitus group

On the basis of continuing to use the results of the 8 participants in Experiment 1, two new tinnitus patients were recruited, and finally, a total of 10 patients participated in the experiment (4 females and 6 males, mean age = 29.4 years). They all had subjective tinnitus (9 of them were bilateral, and one had only right-sided tinnitus) that persisted for more than 6 months, with normal hearing thresholds (≤20 dB HL) at audiometric frequencies from 125 to 8,000 Hz (the only exception was one patient who had a hearing threshold of 30 dB at 8,000 Hz). Institutional Review Board of the Faculty of Psychology, BNU approved the study (202206260076) and all participants provided written informed consents.

##### Normal-hearing adults (NH group)

Ten normal-hearing adults (4 females and 6 males, mean age = 29.4 years) were recruited and matched with each TN group patient for age, sex and education level. They had normal hearing thresholds (≤ 20 dB HL) at audiometric frequencies from 250 to 8,000 Hz (the only exception was one patient who had a hearing threshold of 25 dB at 8,000 Hz), without a history of tinnitus or other hearing disorders. There was no significant group difference between the hearing thresholds of the right ear for both groups, *F*(1, 18) = 4.239, *p* = 0.054. The hearing thresholds of the left ear for the tinnitus group were significantly higher than those for control group, *F*(1, 18) = 5.979, *p* < 0.05. The hear thresholds at left ear for the tinnitus group were significantly higher than those for the control group only at 250 Hz (mean difference: 5.5 dB, *p* < 0.01) and 1,000 Hz (mean difference: 4.5 dB, *p* < 0.05).

#### Stimuli, procedure and data analysis

The material is the same as in Experiment 1. Affected by the COVID-19 epidemic and needing to simplify the experimental process, we used only the stimulus material in which the targets were perceived by participants on the left. The process and data analysis are also the same as in Experiment 1.

#### Design

Experiment 2 had three within-subject factors: masker type (noise masker, speech masker), perceived spatial separation (colocation, separation) and SNRs (four consequent levels from -12, -8, -4, 0, 4 dB). In addition, there was a between-subject factor: group (tinnitus patients, normal-hearing adults). Fifteen target sentences were used in each condition, and 240 trials were used for each participant.

## Results

**FIGURE 2:**
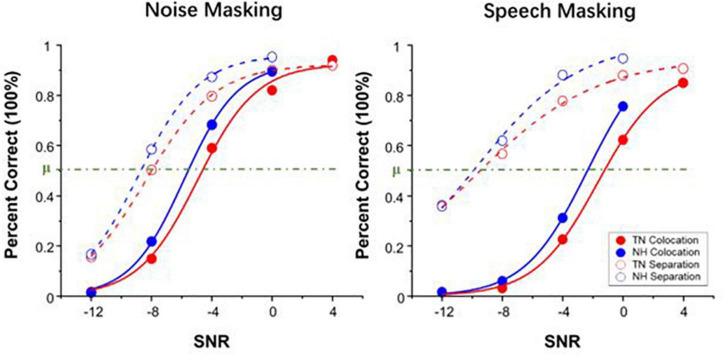
Group mean percent of correct as a function of signal-to-noise ratio (SNR) in noise masking condition **(left panel)** and speech masking condition **(right panel)**. Red and blue represent the data of the TN group and NH group, respectively.

**FIGURE 3:**
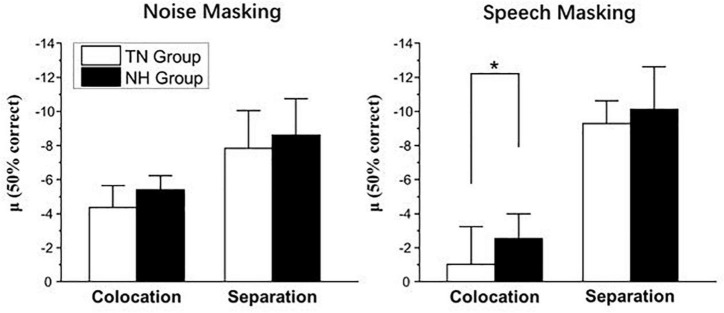
Participants’ SIN perception threshold μ in noise masking condition **(left panel)** and speech masking condition **(right panel)**. *p < 0.05.

Figure 2 shows the group mean percent correct as a function of SNR and the SIN perception threshold (μ, in dB) computed by the psychometric function under two types of masking conditions, noise masking, and speech masking. The thresholds μ are presented separately in Figure 3.

According to Figure 3, a set of independent sample *t*-tests showed that the SIN perception threshold μ of the NH group was significantly lower than that of the TN group only under speech masking’s colocation condition (*t* = 2.460, *p* = 0.024) and marginally significant under noise masking’s colocation condition (*t* = 1.836, *p* = 0.083) but not under noise masking’s separation condition (*t* = 0.926, *p* = 0.367) or speech masking’s separation condition (*t* = 0.689, *p* = 0.500). These results indicate that patients performed significantly worse than normal-hearing peers under speech masking without spatial separation cues and performed as well as their peers under the other conditions.

### Discussion

The results of Experiment 2 found that tinnitus patients perform poorer in SIN perception under speech masking without spatial separation cues than their normal-hearing peers. In fact, people often encounter the situation of recognizing target speech in the scene of multisound source interference, including noise, and speech masking in daily life. In such conditions, listeners were affected by both energetic masking and informational masking so that their speech recognition was more difficult than when masked only by noise. The results of Experiment 2 showed that in the absence of additional perceptual cues, tinnitus patients with normal hearing showed more difficulty under speech masking and little difficulty under noise masking. It can be said that they had weaker performance than normal people only under the most difficult conditions. In addition, patients in the TN group could perform as well as adults in the NH group if they received spatial separation cues in SIN recognition.

The current study distinguished the effects of informational masking and energetic masking well on SIN recognition in patients with tinnitus and normal hearing. Under noise masking conditions, listeners were disturbed only by energetic masking, which affected SIN recognition primarily in the auditory periphery (Freyman et al., 1999; Wu et al., 2005). At this time, the patients’ performances were close to those of normal-hearing adults, indicating that the tinnitus patients did not receive more interference from the auditory periphery. This result matched the patients’ normal pure-tone hearing ability. Informational masking was introduced into the condition of speech masking, and the patients showed significant SIN recognition difficulties compared to normal-hearing adults. This result suggested that tinnitus patients were more disturbed by informational masking than normal adults in the auditory center and higher-level cognitive processing. This result is also consistent with the findings in the study by Cardon et al. (2022) that patients with tinnitus differ from normal adults in cortical auditory evoked potentials, brain signal variability and delayed memory.

There are several putative mechanisms of tinnitus pathophysiology which originated from previous animal research. These mechanisms show that tinnitus is related to “aberrant” neural activity (that is not produced by physically measurable sounds from the environment) that is generated at some level of the auditory system (Cima et al., 2019). In most cases, tinnitus is believed to be associated with some degree of cochlear damage and such damage may not be detected by a standard audiogram. Correspondingly, there are some researchers posing many central mechanisms that can account for the generation of the tinnitus-related activity (Eggermont and Roberts, 2004). However, the central mechanism of tinnitus mostly points to the population with hearing loss, and the central mechanism of tinnitus in these patients with normal hearing is more controversial. In Experiment 2, patients experienced more disturbances in central auditory processing and slightly more peripheral disturbances than their peers. The current study results, to some extent, provide a reference for the etiology or subtype classification of tinnitus patients with normal hearing. Their performance suggested that their SIN recognition difficulties originate primarily in the auditory centers, which are more inclined to suggest that tinnitus patients have the influence of related activities at the central level.

There did not appear to be a decline in tinnitus patients’ ability to use spatial separation cues. With the help of cognitive cues, their speech recognition performance can be as good as that of normal-hearing adults. This suggests that patients with tinnitus are more dependent on cognitive cues to some extent. Most of the time, they do not experience many distractions or are aided by enough cognitive cues, and their speech recognition performance can be unaffected by tinnitus. However, in some difficult auditory situations, they may show more difficulty than normal-hearing peers.

## General discussion

The current study considered interaural differences in the speech perception performance of tinnitus patients with normal hearing under noise masking and speech masking with spatial separation or not in Experiment 1 and compared the performance between tinnitus patients and normal-hearing adults in Experiment 2. First, we found no interaural differences in tinnitus patients in each condition, refuting the effects of tinnitus with lateralization. Then, we found that tinnitus patients had significantly more difficulties in SIN recognition than normal-hearing adults under speech masking without spatial separation cues but performed as well as normal people with spatial separation cues.

The manifestations of normal-hearing tinnitus patients are similar to those of other hearing-difficulty groups, such as elderly individuals (Anderson et al., 2013; Zhang et al., 2021) and cured ISSHL patients (Diao et al., 2022), who experience difficulties under both types of masking without spatial cues and could release from masking in SIN recognition to a great extent *via* cognitive cues. From the perspective of the auditory processing mechanism, this again proved that SIN, especially in reverberant noisy environments, was a complex cognitive process including sensory input from the auditory periphery and top-down cognitive processing. Our experiments considered the distinction between energy masking and information masking and found that the increased disturbances in these patients did not come from the periphery. Whether the increased disturbances they receive at higher levels come from tinnitus’ integration of attention, speech recognition strategies, or the integration of the auditory center can be investigated in future studies.

In addition, in some previous studies, the age range of the tinnitus group was larger, and there were some elderly tinnitus groups, so researchers have explored age-related tinnitus (Heeren et al., 2014; Tai and Husain, 2018, 2019; Zeng et al., 2020). However, the effect of aging seems uncertain in the tinnitus population because the effects of aging on cognitive processing or control and on auditory processing are almost concomitant. The participants in our study were all under the age of 40 and belonged to a younger group, so we could relatively simply reflect the effect of tinnitus on SIN recognition processing and ignore the age factor. The present results provide a good indication that tinnitus causes speech recognition difficulties in patients, although it does not produce pure-tone hearing impairment. In follow-up studies of the auditory processing mechanism of tinnitus patients, it may be considered to include elderly tinnitus patients for age grouping and to use experimental techniques that better reflect the ability of auditory center integration (for example, functional brain imaging technology). Additionally, the study participant population was fixed as chronic normal-hearing tinnitus patients. In fact, tinnitus symptoms have many possibilities, acute or chronic, and a complex relationship with hearing impairment (Ma et al., 2021).

## Data availability statement

The raw data supporting the conclusions of this article will be made available by the authors, without undue reservation.

## Ethics statement

The studies involving human participants were reviewed and approved by Institutional Review Board of the Faculty of Psychology, BNU. The patients/participants provided their written informed consent to participate in this study.

## Author contributions

MW contributed to the design of the study, the analysis, interpretation of the data, and drafting the manuscript. JL contributed to the acquisition, analysis of the data, and the writing of the manuscript. LK contributed to the interpretation of the data and the final version of the manuscript. YZ contributed to the recruitment of participants and theoretical support for tinnitus symptoms. TD contributed to the recruitment of participants and reviewed the final draft very carefully. XM contributed to the theoretical support for tinnitus symptoms. All authors contributed to the article and approved the submitted version.
